# *Retracted*: Connexin 43 upregulation by dioscin‐inhibited gastric cancer metastasis by suppressing PI3K/Akt pathway

**DOI:** 10.1002/fsn3.3006

**Published:** 2022-08-17

Retraction: Kou, Y., Zhu, R., Sun, Q., Li, Z., Feng, X., & Xu, H. (2022). Connexin 43 upregulation by dioscin‐inhibited gastric cancer metastasis by suppressing PI3K/Akt pathway. *Food Science & Nutrition*. https://doi.org/10.1002/fsn3.3006. The above article from *Food Science & Nutrition*, published online on 17 August 2022 in Wiley Online Library (https://wileyonlinelibrary.com), has been retracted by agreement between the journal's editor‐in‐chief, Y. Martin Lo, and Wiley Periodicals LLC. This action has been agreed upon due to image manipulation in Figures 1 and 4. As a result, the findings of this manuscript are considered unreliable. The editorial office did not receive responses from the authors or the institute.

